# PLGA-encapsulated CpG ODN and *Campylobacter jejuni* lysate modulate cecal microbiota composition in broiler chickens experimentally challenged with *C*. *jejuni*

**DOI:** 10.1038/s41598-018-30510-w

**Published:** 2018-08-13

**Authors:** Khaled Taha-Abdelaziz, Alexander Yitbarek, Tamiru Negash Alkie, Douglas C. Hodgins, Leah R. Read, J. Scott Weese, Shayan Sharif

**Affiliations:** 1 Department of Pathobiology, Ontario Veterinary College, University of Guelph, Guelph, ON N1G 2W1 Canada; 2 Pathology Department, Faculty of Veterinary Medicine, Beni-Suef University, Al Shamlah, Beni-Suef, 62511 Egypt; 3 Present Address: Department of Biology, Wilfred Laurier University, Waterloo, ON N2L 3C5 Canada

## Abstract

*Campylobacter jejuni* is a leading bacterial cause of human gastroenteritis. Reducing *Campylobacter* numbers in the intestinal tract of chickens will minimize transmission to humans, thereby reducing the incidence of infection. We have previously shown that oral pre-treatment of chickens with *C*. *jejuni* lysate and Poly D, L-lactide-co-glycolide polymer nanoparticles (PLGA NPs) containing CpG oligodeoxynucleotide (ODN) can reduce the number of *C*. *jejuni* in infected chickens. In the current study, the effects of these pre-treatments on the composition and functional diversity of the cecal microbiota, in chickens experimentally infected with *C*. *jejuni*, were investigated using next-generation sequencing. The taxonomic composition analysis revealed a reduction in cecal microbial diversity and considerable changes in the taxonomic profiles of the microbial communities of *C*. *jejuni*-challenged chickens. On the other hand, irrespective of the dose, the microbiota of PLGA-encapsulated CpG ODN- and *C. jejuni* lysate-treated chickens exhibited higher microbial diversity associated with high abundance of members of Firmicutes and Bacteroidetes and lower numbers of *Campylobacter* than untreated-chickens. These findings suggest that oral administration of encapsulated CpG ODN and *C*. *jejuni* lysate can reduce colonization by *C*. *jejuni* by enhancing the proliferation of specific microbial groups. The mechanisms that mediate these changes remain, however, to be elucidated.

## Introduction

Even though microbial communities of the intestine have long been known to influence poultry growth and health, the taxonomic and functional characterization of these communities have only recently begun to be fully studied. Recent advances in high-throughput next generation sequencing technologies have contributed to rapid progress in this field. The gut microbiota is described as a complex and dynamic ecosystem formed by diverse microbial communities^1^. In chickens, the development of gut microbiota commences immediately after hatch and is established within the first weeks of life^2,3^. The cecum is reported to be the most densely populated region of the gut due to the relatively low oxygen partial pressure, gut enzymes, and concentrations of bile acids and salts^4^. It harbors a highly diversified microbial community, including *Bacteroides*, *Bifidobacterium*, *Clostridium*, *Enterococcus*, *Escherichia*, *Fusobacterium*, *Lactobacillus*, and *Streptococcus*, with densities reaching 10^12^ per gram of luminal content^5–7^. *Campylobacter jejuni* is also considered a commensal bacterium of the chicken intestine. It colonizes the chicken intestine by 3 weeks of age at densities of 10^8^ colony forming units (CFUs) per gram of cecal content and infected chickens become carriers and can shed the bacterium for the rest of their life^8^. Even though this bacterium does not cause clinical disease in chickens^9^, consumption of undercooked poultry meat, contaminated by intestinal contents, or contact with surfaces or items contaminated by *C*. *jejuni* may lead to foodborne illness in humans^10^. Although biosecurity measures can reduce on-farm colonization with *C*. *jejuni*, complementary control measures are needed to reduce infection rates^11^. Numerous vaccination strategies have been evaluated for the control of *C*. *jejuni* in chickens; however, none of these strategies demonstrated sufficient protection.

Enhancing anti-bacterial mucosal immune responses of chicks by oral vaccination or prophylactic use of antimicrobial alternatives has demonstrated considerable promise to reduce enteric *Campylobacte*r colonization^12,13^. However, despite the practicality of these approaches, their interaction with the mucosal immune system and/or luminal microbial communities of the gut may result in changes in the composition or function of gut microbiota^14,15^. For example, considerable changes were observed in the cecal microbial ecology of chickens orally vaccinated with *Salmonella* vaccines^14,15^. Recent evidence has demonstrated that drastic alterations in the microbiota composition can result in dysregulation of host immune homeostasis^16,17^. Since the stability of the gut microbial ecology is important in maintaining intestinal homeostasis^6,7,17^ and there can be far reaching impacts of alternation of the gut microbiota on other body systems, the effects of oral *C*. *jejuni* vaccines on gut microbiota should be investigated.

Analysis of the gut microbiota can clarify its role in enteric diseases^18^, and also help explain the broad protection conferred by some vaccines and dietary intervention strategies. For instance, Thibodeau and colleagues have demonstrated a reduction in relative abundance of *Streptococcus* in the cecal microbiota of chicken when essential oils were used as feed additives to control *C*. *jejuni*^12^. We have recently demonstrated that oral administration of PLGA-encapsulated CpG ODN (Poly (D,L-lactide-co-glycolide) polymer nanoparticles (PLGA NPs) containing CpG oligodeoxynucleotide (ODN) as well as *C*. *jejuni* lysate can reduce the intestinal burden of *C*. *jejuni* in broiler chickens^13^. The protective contribution of intestinal and systemic immune responses has been demonstrated^13,19^; however, the impacts of these treatments on gut microbial communities have not been investigated. Therefore, the present study was undertaken to characterize the composition of the cecal microbiota following oral delivery of PLGA-encapsulated CpG ODN as well as *C*. *jejuni* lysate in broiler chickens experimentally challenged with *C*. *jejuni*.

## Results

**Figure 1:**
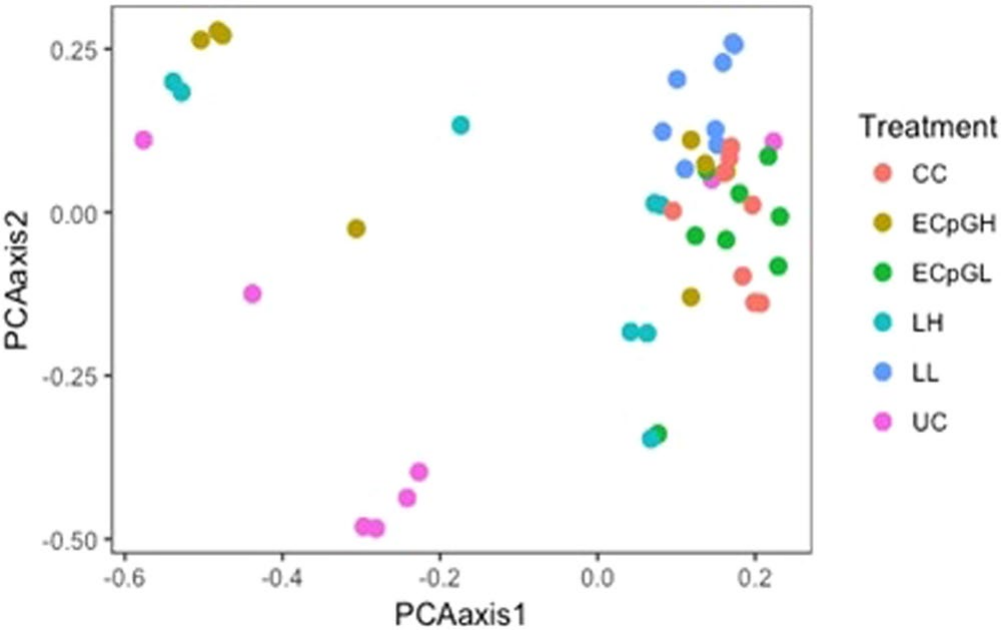
Non-metric multi-dimensional scaling (NMDS) plot illustrating the chicken cecal microbiota beta-diversity of all groups (PBS-treated, *C*. *jejuni*-challenged (CC) chickens, low (ECpGL) and high dose (ECpGH) of PLGA-encapsulated CpG-ODN-treated, low (LL) and high dose (LH) of *C*. *jejuni* lysate-treated, and unchallenged control (UC) chickens). Chickens were treated orally at 14 days post-hatch with a low (5 µg) or high dose (25 µg) of encapsulated CpG ODN, or a low or high dose of *C*. *jejuni* lysate, or received PBS as a placebo, and were orally challenged with 10^7^ CFUs of *C*. *jejuni* at 15 days post-hatch. At 22 days post-challenge with *C*. *jejuni*, chickens were necropsied and cecal contents were collected for analysis. *P* < 0.05 was considered significant.

In the present study, the impacts of *C*. *jejuni* infection as well as pre-treatments with either encapsulated CpG ODN (ECpG) or *C*. *jejuni* lysate on cecal microbiota were investigated. In general, alpha diversity analysis revealed greater microbial richness in groups treated with a low dose of encapsulated CpG (ECpGL) or a high dose of *C*. *jejuni* lysate (LH) compared to the *C*. *jejuni*-challenged group. With respect to beta diversity, non-metric multi-dimensional scaling (NMDS) plot showed a distinct separation among treatments with a high dose of encapsulated CpG (ECpGH) or LH treatments showing diversification appears as a scattered population of microbiota compared to *C*. *jejuni*-challenged (Fig. 1).

### The impact of *Campylobacter* infection on cecal microbiota

**Figure 2:**
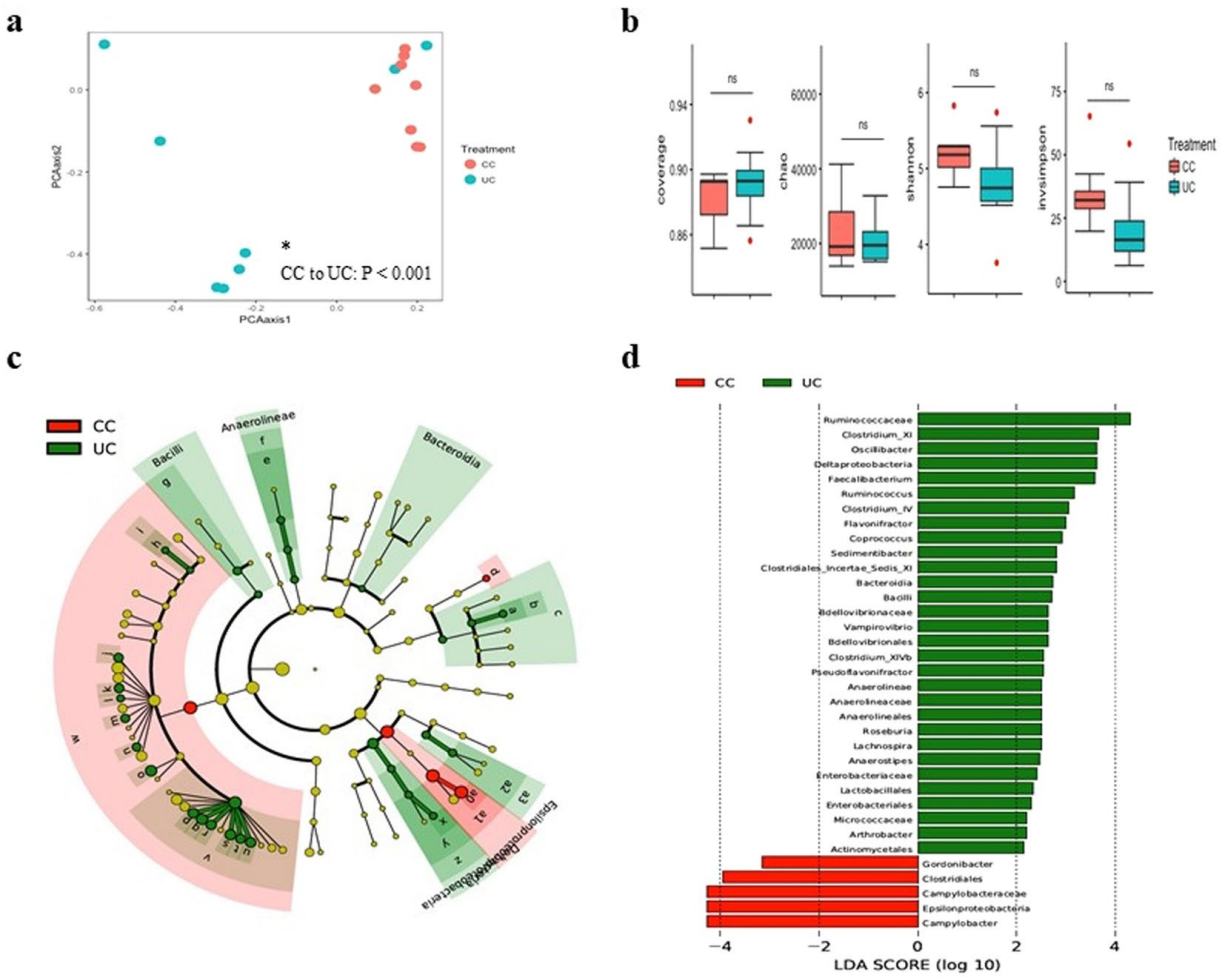
Comparison of cecal microbiota of PBS-treated *C*. *jejuni*-challenged (CC) and unchallenged control (UC) chickens. **(a)** Non-metric multi-dimensional scaling (NMDS) plot illustrating the chicken cecal microbiota beta-diversity. (a*) Analysis of molecular variance (AMOVA) where p-values demonstrating significant differences in the NMDS plot, *P* < 0.05 was considered significant. **(b)** Alpha diversity analysis illustrating species richness and abundance. **(c)** LEfSe cladogram and **(d)** Linear discriminant analysis (LDA, horizontal bars) illustrating differential enrichment of microbes. Chickens of the CC group were treated orally at 14 days post-hatch with PBS as a placebo and were orally challenged with 10^7^ CFUs of *C*. *jejuni* at 15 days post-hatch. At 22 days post-challenge with *C*. *jejuni*, chickens were necropsied and cecal contents were collected for analysis.

Alpha diversity analysis on the effect of *C*. *jejuni* challenge on the species richness and diversity of cecal microbial communities showed no significant differences (P > 0.05) (Fig. 2b). Beta diversity analysis showed that the unchallenged group with the chicken’s microbial community appearing as a scattered population while *C*. *jejuni-*challenged group clustered together (Fig. 2a), and the AMOVA analysis showed that *C*. *jejuni* challenge significantly affected the cecal microbial communities of chickens. Analysis of NMDS of PBS-treated *C*. *jejuni-*challenged (CC) and unchallenged (UN) groups showed a distinct separation of treatments (AMOVA, p < 0.001, Fig. 2a). Comparison of differential enrichment of the gut microbiota of unchallenged to *C*. *jejuni*-challenged chickens using LEfSe showed that infected chickens were highly enriched with order *Clostridiales* and genera *Campylobacter* and *Gordonibacter*. The gut microbiota of unchallenged chickens was significantly enriched with class *Deltaproteobacteria*, *Bacteroidia* and *Bacilli*, order *Bdellovibrionales*, *Anaerolineales*, *Lactobacillales* and *Actinomycetales*, family *Ruminococcaceae*, *Clostridiales_Incertae_Sedis* cluster XI, *Bdellovibrionaceae*, *Anaerolineaceae*, *Enterobacteriaceae* and *Micrococcaceae*, and genera *Clostridium* cluster XI, *Oscillibacter*, *Faecalibacterium*, *Ruminococcus*, *Clostridium* cluster IV, *Flavonifractor*, *Coprococcus*, *Sedimentibacter*, *Vampirovibrio*, *Clostridium* cluster XlVb, *Pseudoflavonifractor*, *Anaerolineae*, *Roseburia*, *Lachnospira*, *Anaerostipes and Arthrobacter*, all at an LDA score of above 2.0 (Fig. 2c,d). Furthermore, this was further confirmed by analysis of significant changes in the relative abundance (Kruskal-Wallis tests, FDR-adjusted Q < 0.05; Fig. S1a,b).

### The impact of encapsulated CpG ODN on cecal microbiota of chickens infected with *C*. *jejuni*

**Figure 3:**
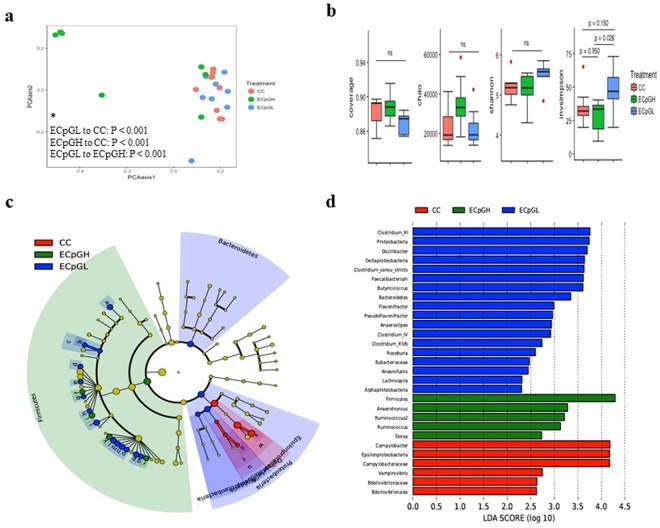
Comparison of cecal microbiota of low (ECpGL) and high dose (ECpGH) of PLGA-encapsulated CpG-ODN-treated and PBS-treated, *C*. *jejuni*-challenged (CC) chickens. **(a)** Non-metric multi-dimensional scaling (NMDS) plot illustrating the chicken cecal microbiota beta-diversity. (a*) Analysis of molecular variance (AMOVA) where p-values demonstrating significant differences between treatments, *P* < 0.05 was considered significant. **(b)** Alpha diversity analysis illustrating species richness and abundance. **(c)** LEfSe cladogram and **(d)** Linear discriminant analysis (LDA, horizontal bars) illustrating differential enrichment of microbes. Chickens were treated orally at 14 days post-hatch with a low (5 µg) or high dose (25 µg) of encapsulated CpG ODN, or received PBS as a placebo and were orally challenged with 10^7^ CFUs of *C*. *jejuni* at 15 days post-hatch. At 22 days post-challenge with *C*. *jejuni*, chickens were necropsied and cecal contents were collected for analysis. *P* < 0.05 was considered significant.

Analysis of Inverse Simpson measure of alpha-diversity index showed that the group received ECpGL exhibited a significantly higher microbial richness compared to the group received ECpGH (P = 0.026) (Fig. 3b). Analysis of NMDS of both ECpGL and ECpGH administered and *C*. *jejuni*-challenged chickens showed a distinct separation among treatments with ECpGH treatment showing diversification appears as a scattered population of microbiota compared to *C*. *jejuni*-challenged and ECpGL (Fig. 3a) and the AMOVA test revealed significantly different cecal microbial communities among treatments (p < 0.001, Fig. 3a). The differential enrichment of the gut microbiota of chickens after ECpG ODN administration, either at a lower or higher dose, using LefSe, showed that the *C*. *jejuni*-challenged group (CC) was enriched with genus *Campylobacter* and *Vampirovibrio*, while the group treated with low dose of ECpG was enriched with phylum Proteobacteria and Bacteroidetes, class *Alphaproteobacteria* and *Deltaproteobacteria*, family *Eubacteriaceae*, genus *Oscillibacter*, *Clostridium_sensu_stricto*, *Faecalibacterium*, *Butyricicoccus*, *Flavonifractor*, *Pseudoflavonifractor*, *Anaerostipes*, *Clostridium* cluster IV, *Clostridium* cluster XIVb, *Roseburia*, *Anaerofustis*, *Lachnospira* and *Clostridium* cluster XI. The group with high dose of ECpG was highly enriched with phylum Firmicutes, genus *Anaerotruncus*, and two *Ruminococcus* and *Ruminococcus* spp, all at an LDA score of >2.0 (Fig. 3c,d). Significantly higher relative abundance of the genera two *Ruminococcus*, *Clostridium* cluster XIVb, *Blautia* and *Clostridium* cluster XIVa were observed in CpGL compared to both CC and CpGH (Kruskal-Wallis tests, FDR-adjusted Q < 0.05; Fig. S2a,b).

### The impact of *C*. *jejuni* lysate on cecal microbiota of chickens infected with *C*. *jejuni*

**Figure 4:**
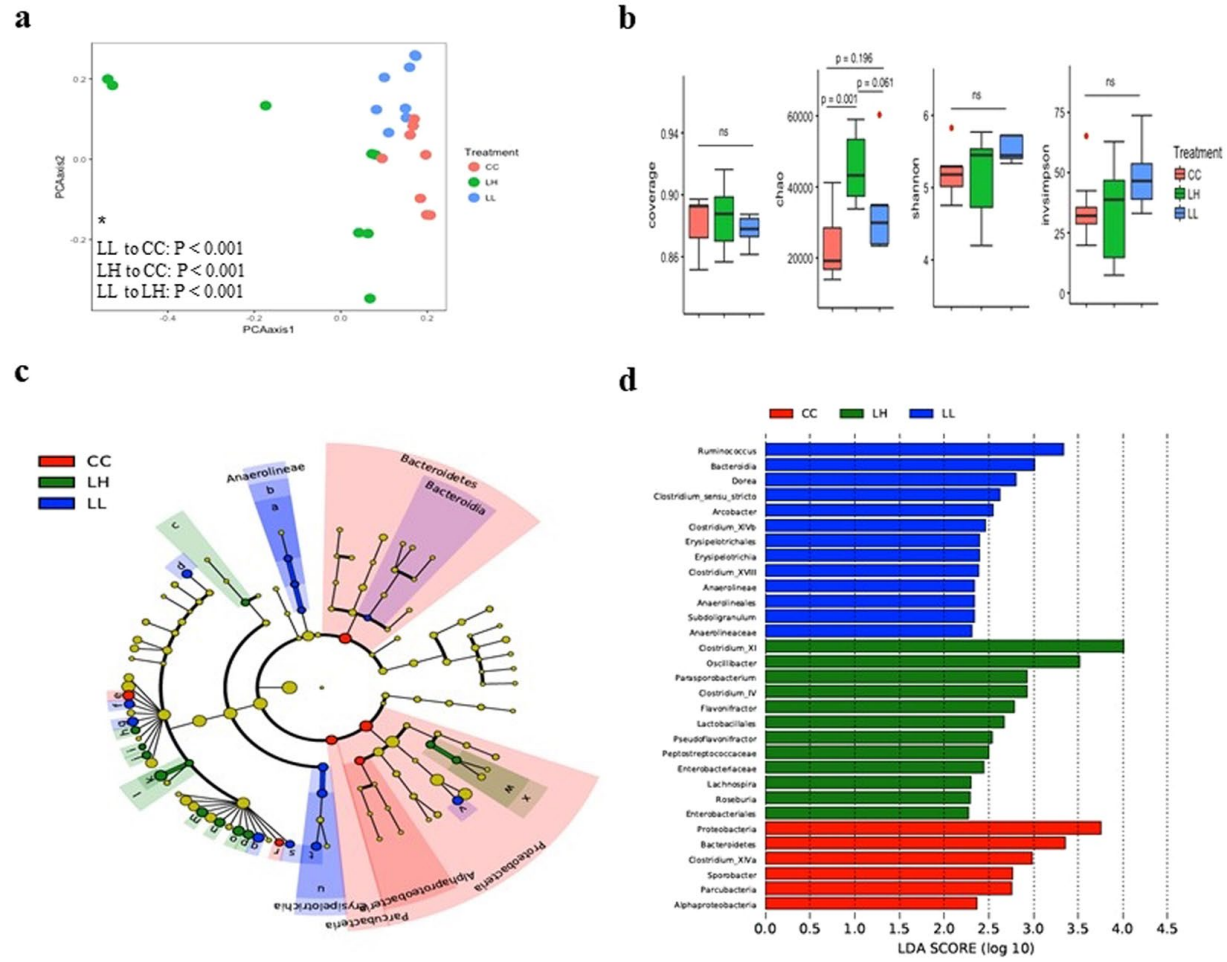
Comparison of cecal microbiota of low (LL) and high dose (LH) of *C*. *jejuni* lysate-treated and PBS-treated, *C*. *jejuni*-challenged (CC) chickens **(a)** Non-metric multi-dimensional scaling (NMDS) plot illustrating the chicken cecal microbiota beta-diversity. (a*) Analysis of molecular variance (AMOVA) where p-values demonstrating significant differences between treatments, *P* < 0.05 was considered significant. **(b)** Alpha diversity analysis illustrating species richness and abundance. **(c)** LEfSe cladogram and **(d)** Linear discriminant analysis (LDA, horizontal bars) illustrating differential enrichment of microbes. Chickens were treated orally at 14 days post-hatch with a low or high dose of *C*. *jejuni* lysate, or received PBS as a placebo and were orally challenged with 10^7^ CFUs of *C*. *jejuni* at 15 days post-hatch. At 22 days post-challenge with *C*. *jejuni*, chickens were necropsied and cecal contents were collected for analysis. *P* < 0.05 was considered significant.

Alpha diversity analysis showed that the group that received high dose *C. jejuni* lysate (LH) had a significantly higher Chao1 diversity index (P = 0.001) compared to the *C*. *jejuni*-challenged group (CC), indicating greater microbial richness (Fig. 4b). Analysis of NMDs of chickens in low dose of *C*. *jejuni* lysate (LL) and LH groups showed a distinct separation among treatments with LH treatment showing diversification of the microbial community appears as a scattered population of microbiota compared to *C*. *jejuni*-challenged and LL (Fig. 4a), and AMOVA analysis showed significantly different cecal microbial communities among treatments (p < 0.001, Fig. 4a). The differential enrichment of the gut microbiota of chickens using LefSe showed that the CC group was enriched with phylum Proteobacteria, Bacteroidetes and Parcubacteria, class *Alphaproteobacteria*, and genus *Clostridium* cluster XIVa and *Sporobacter*. The group with low dose of *C*. *jejuni* lysate was enriched with class *Bacteroidia*, and *Erysipelotrichia*, order *Erysipelotrichales* and *Anaerolineales*, family *Anaerolineaceae*, genus *Ruminococcus*, *Dorea*, *Clostridium sensu stricto*, *Arcobacter*, *Clostridium* cluster XIVb, *Clostridium* cluster XVIII, *Anaerolineae* and *Subdoligranulum*. The group with high dose of *C*. *jejuni* lysate was highly enriched with order *Lactobacillale*s and *Enterobacteriales*, family *Peptostreptococcaceae* and *Enterobacteriaceae*, and genus *Clostridium* cluster XI, *Oscillibacter*, *Parasporobacterium*, *Clostridium* cluster IV, *Flavonifractor*, *Pseudoflavonifractor*, *Lachnospira* and *Roseburia*, all at an LDA score of >2.0 (Fig. 4c,d). These observations were also further confirmed by analysis of significant changes in the relative abundance at the genus level as shown in Fig. S3a,b.

## Discussion

The bidirectional interactions between the host immune system and the microbiota are crucial for maintaining normal intestinal homeostasis. Accumulating evidence supports a role for intestinal mucosal immunity in shaping and regulating the composition of the intestinal microbiota and vice versa^20^. Whilst our recent findings indicated that ECpG ODN and *C*. *jejuni* lysate can elicit intestinal innate responses when administered orally in broiler chicks^19^, the impact of these treatments on the intestinal microbial ecology in chickens experimentally infected with *C*. *jejuni* had not been evaluated. Therefore, the current study was undertaken to investigate the effects of these treatments on the composition and functional diversity of the cecal microbiota of chickens challenged with *C*. *jejuni*. In general, our results revealed dynamic changes in the microbial community of treated chickens; the microbiota of ECpG ODN- and *C. jejuni* lysate-treated chickens had higher microbial diversity and lower numbers of *Campylobacter* than that of challenged untreated-chickens, suggesting a possible recovery in the microbial composition. These results confirm and extend our earlier findings indicating that oral administration of ECpG ODN and *C*. *jejuni* lysate reduces the intestinal burden of *Campylobacter* in broiler chickens^19^.

Despite the fact that *Campylobacter* is considered a member of normal gut microbiota in chickens^21,22^, intestinal colonization with this bacterium has been shown to be associated with changes in taxonomic composition of the microbiota^12,23^. The results of the present study are broadly consistent with these observations; compositional analysis revealed a reduction in cecal microbial diversity and considerable changes in the taxonomic profiles of the microbial communities of *C*. *jejuni*-challenged chickens with higher abundance of genus *Gordonibacter*, *Campylobacter*, and order *Clostridiales* and lower proportions of *Blautia*, *Clostridium* cluster XlVa, *Clostridium* cluster XlVb, *Ruminococcus*, *Ruminococcus*, *Sporacetigenium*, *Pseudoflavonifractor*, and *Dorea*, compared to the unchallenged chickens. However, it is unclear whether these compositional changes and the dominance of *Campylobacter* are due to the direct ability of *C*. *jejuni* to displace indigenous species, a phenomenon called competitive exclusion, or are related to the impact of metabolic by-products of the bacterial species associated with *Campylobacter* colonization. It is well established that gut bacterial metabolites can contribute to the colonization of specific microbial groups^24^. In a rat model, it has been shown that *Gordonibacter* species are capable of metabolizing ellagic acid to urolithin-A (3,8-dihydroxybenzo[c]chromen-6-one)^25^, which, in turn, alters microbial community, probably due to its antimicrobial activity^26^. Thus, when considering the role of gut microbiota in regulating the host defense against enteric infection, it is likely that the alterations in the microbial composition may influence colonization resistance to *C. jejuni*^27,28^.

Numerous studies have shown that manipulation of gut microbiota may help preclude colonization by *C*. *jejuni*. For example, dietary supplementation with short chain organic acids and phenolic essential oils modified the gut microbiota^29^ associated with reduction in *C*. *jejuni* colonization^12^. In view of this, we have demonstrated that oral administration of the high dose of ECpG ODN leads to an increase in microbial diversity and in the relative abundance of members of phylum Firmicutes, particularly genera *Clostridium* and *Ruminococcus*, with a concomitant decline in *Campylobacter* numbers. Similarly, LefSe analysis revealed that chickens treated with either low or high doses of *C*. *jejuni* lysate exhibited lower levels of Proteobacteria (this phylum includes the genera *Campylobacter*, *E*. *coli*, *Salmonella*, *Vibrio*, *Helicobacter*, and *Yersinia*), and a diverse cecal microbiota dominated by members of phyla Firmicutes and Bacteroidetes. Indeed, the cecal microbiota of high *C*. *jejuni* lysate group was enriched with *Clostridium* cluster XI, *Oscillibacter*, *Parasporobacterium*, *Clostridium* cluster IV, *Lactobacillales Pseudoflavonifractor*, *Peptostreptococcaceae*, *Lachnospira*, *and Roseburia* (all belong to phylum Firmicutes), and genus *Flavonifractor* (in the phylum Bacteroidetes), whereas the microbiota of low *C*. *jejuni* lysate was enriched with *Ruminococcus*, *Erysipelotrichales*, *Erysipelotrichia*, *Clostridium* cluster XVIII, *Clostridium* cluster XlVb, *Clostridium sensu stricto*, *Subdoligranulum*, *Dorea* (all belonging to the phylum Firmicutes) and class *Bacteroidia* (in the phylum Bacteroidetes).

A previous study reported that the gut microbiota of healthy broiler chickens is dominated by two main phyla, Firmicutes and Bacteroidetes^30^. Strikingly, irrespective of the dose, treatment with ECpG ODN or *C*. *jejuni* lysate has shown great potential to restore the microbial composition to its normal status, even though the mechanisms that drive these changes remain to be investigated. The cecal microbial shifts towards elevated Firmicutes and the absence or low abundance of phylum Proteobacteria, in both ECpG ODN and *C*. *jejuni* lysate groups, indicates a clear association between the abundance of Firmicutes and colonization by *C*. *jejuni* as well as the recovery of the gut microbiota from a dysbiotic condition after *C*. *jejuni* challenge. These observations were corroborated by a recent study in mice that showed that colonization with low numbers of Firmicutes was associated with decreased resistance to colonization by *C*. *jejuni*^27^. Another study in humans reported that the high abundance of genera *Bacteroides* and *Prevotella* and *Ruminococcus* with the absence or low abundance of the phylum Proteobacteria is indicative of a healthy gut microbiota^31^.

In addition to the effects of intestinal immune responses initiated by treatment with ECpG ODN and *C*. *jejuni* lysate^19^, the lower numbers of *Campylobacter* in the cecal contents of these treatment groups could also be attributed to the dominance of mucin-fermenting bacteria, particularly the genera *Ruminococcus*, *Clostridium* and *Bacteroides*. These bacteria have been shown to have the potential to ferment and degrade the mucous layer covering the intestinal epithelium^32,33^, allowing cross-talk of *Campylobacter* and epithelial cells as well as the direct exposure of *Campylobacter* to CpG ODN- and *C*. *jejuni* lysate-induced antimicrobial peptides^19^. Another possible explanation of lower cecal populations of *Campylobacter* lies in the increased abundance of lactic acid-producing bacteria, such as *Lactobacillus* spp. (in the high dose *C*. *jejuni* lysate group) and butyrate-producing clusters of Firmicutes, in particular *Ruminococcus* and *Clostridium* cluster IV, cluster XIVa, and cluster XIVb (in both ECpG ODN and *C*. *jejuni* groups) and the consequent increase in lactic acid and butyrate levels in the gut. Indeed, butyrate has recently emerged as a key metabolite in the maintenance of gut health^34^. In addition to its role in reducing the pathogenicity of enteric pathogens^34^, inclusion of butyrate in the chicken diet has been shown to reduce *Campylobacter* burden in the gut^35^, probably through induction of antimicrobial peptides in the gastrointestinal tract^36^. Oral administration of *Lactobacillus* spp. has also shown potential to reduce cecal colonization by *C*. *jejuni* in chickens. Several mechanisms have been proposed for anti-*Campylobacter* activity of *Lactobacillus*, including elicitation of both innate and adaptive immune responses^37^, production of organic acids such as lactic acid and alteration of microbiota composition^38^. We have recently obtained evidence that *Lactobacillus* spp. possess bactericidal activity against *C*. *jejuni* when co-cultured *in vitro* (Taha-Abdelaziz *et al*., unpublished data). It is, therefore, tempting to speculate that the enrichment of mucin-fermenting bacteria or lactic acid- and butyrate-producing bacteria in the ceca of ECpG ODN and *C*. *jejuni* lysate groups mediates colonization resistance against *C*. *jejuni*.

In conclusion, this study describes the impact of oral administration of ECpG ODN and *C*. *jejuni* lysate on cecal microbial community patterns of *Campylobacter-*infected chickens. The role of ECpG ODN and *C*. *jejuni* lysate has been shown to extend beyond their direct immunostimulatory activity^19^ as they have demonstrated potential to restore intestinal microbial balance. Furthermore, our findings suggest that ECpG ODN and *C*. *jejuni* lysate can help preclude colonization by *C*. *jejuni* through enhancing the proliferation of specific microbial groups, in particular phyla Firmicutes and Bacteroidetes; however, the mechanisms by which ECpG ODN and *C*. *jejuni* lysate shift the microbial composition that resulted in reduced *C*. *jejuni* remains to be elucidated. Nevertheless, these findings pave the way for further research on the abundance of functional gene and fermentation profiles of these microbial communities.

## Materials and Methods

### Chickens

One-day-old commercial broiler chicks (Ross 708, Maple Leaf Foods, New Hamburg, Ontario) were randomly assigned to floor pens with dry, clean wood shavings. Chickens were fed antibiotic- and feed additive-free diets ad libitum. During the experimental period, chickens were monitored for general health problems. Chickens remained healthy throughout the experiment. This research was approved by the University of Guelph Animal Care Committee in compliance with the guidelines of the Canadian Council on Animal Care.

### Preparation of *Campylobacter* culture and lysate

*Campylobacter jejuni* strain 81–176 was cultured as previously described^39^. Briefly, a loop of frozen *C*. *jejuni* glycerol stock was streaked onto Columbia Agar with 5% Sheep Blood or Mueller–Hinton (MH) agar (Oxoid, Basingstoke, Hampshire, UK) and incubated for 18 h at 41 °C under microaerophilic conditions of 10% CO_2_, 5% O_2_, and 85% N_2_. Subsequently, several colonies of *C*. *jejuni* were inoculated into 100 mL fresh Mueller–Hinton broth and incubated at 41 °C under microaerophilic conditions to reach mid-log phase (determined by growth curve analysis). A mid-log culture of *C*. *jejuni* was centrifuged and washed with Dulbecco phosphate-buffered saline (DPBS), and diluted in DPBS to an OD 600 nm of 0.01 which corresponds to approximately 2.0 × 10^7^ CFU/ml. The suspended bacteria were lysed as previously described^13^. Briefly, the bacteria were heat-killed at 65 °C for 30 min and then sonicated on ice (six 15-s pulses interspersed with 30-s pauses). The protein concentration was measured using a BCA Protein Assay Kit (Thermo Fisher Scientific, Rochester, USA). One milliliter of lysate (2.0 × 10^7^ CFU/ml) contained approximately 8.6 µg protein. The lysate was stored at −80 °C until use.

### CpG ODN

A synthetic class B CpG ODN 2007 [5′-TCGTCGTTGTCGTTTTGTCGTT-3′], with a phosphorothioate backbone, was purchased from Sigma, St. Louis, USA. CpG-loaded PLGA NPs were prepared by the method of water-oil-water double emulsion, solvent evaporation method as described previously^40^. Briefly, CpG ODN was allowed to form a complex with polyethylenimine at a molar ratio of 5 (ratio of primary amino in polyethylenimine to phosphate in ODNs) and stirred at intervals. The resulting complex was ultrasonicated with 4.5% cold PLGA (PLGA dissolved in dichloromethane) for 1 minute (Ultrasonic processor, 3 mm probe diameter, Fisher Scientific, Ottawa, ON, Canada). The primary emulsion was further sonicated for 2 min in 2% Poly(vinyl) alcohol (PVA). The resulting emulsion was immediately transferred into nanoparticle hardening media containing 2% PVA. The nanoparticles were collected after several washings to remove excess PVA and were lyophilized.

The physicochemical properties of the nanoparticles such as size, polydispersity index, zeta potential (surface charge) and solubility were determined as previously described^41^. Dynamic light scattering analysis indicated that PLGA NPs encapsulating CpG ODN had an average diameter of 533 nm in liquid non-buffered media with a net surface charge of −15 mV. Moreover, the particles had a polydispersity index of 0.095 that measure the width of size distributions of the particles. The lower zeta value of PLGA NPs encapsulating CpG ODN was expected as traces of polyethylenimine, used to form complexes with CpG ODN were adsorbed on the surface of the particles.

The encapsulation of CpG ODN in PLGA NPs was determined after re-suspending 1 mg of lyophilized nanoparticles in TE buffer. The resulting solution was further dissolved in dichloromethane for 1 hr. The suspension was centrifuged at 9000 xg for 5 minutes and the supernatant was collected to quantify CpG ODN using Quant-iT™ OliGreen® ssDNA reagent and kit system (Invitrogen) and a GloMax® -Multi Detection System Fluorometer (Promega, Madison, WI). The encapsulation efficiency for CpG ODN was 69%. The nanoparticles were found readily monodispersed in water (pH 5) and PBS (pH 7.4) at a concentration between 1 mg/mL to 20 mg/mL.

### Immunization and challenge

Forty-eight one-day-old broiler chicks were randomly divided into 6 groups. At 14 days of age, chickens were orally treated with a low dose (5 µg) of encapsulated CpG ODN (ECpGL), or a high dose (25 µg) of ECpG ODN (ECpGH), or a low dose (4.3 µg protein) of *C*. *jejuni* lysate (LL), or a high dose (21.5 µg protein) of *C*. *jejuni* lysate (LH), or PBS (*C*. *jejuni*-challenged, CC) 24 h before oral challenge with 10^7^ CFUs of *C*. *jejuni*. The unchallenged, untreated control group (UC) was kept in a separate room. Cecal contents were collected (n = 8 per group) at 22 days post-infection (comparable to slaughter age for broilers) (Table 1).

**Table 1:** Experimental design.

Group	Number of birds	Treatment	Treatment day	Challenge dose and day	Sampling day
1- (UC)	8	—	—	—	37 days of age
2- (CC)	8	PBS	14 days of age	10^7^ CFUs of *C*. *jejuni* 15 days of age	37 days of age (22 days post-challenge)
3- (ECpGL)	8	5 µg ECpG ODN	14 days of age	10^7^ CFUs of *C*. *jejuni* 15 days of age	37 days of age (22 days post-challenge)
4- (ECpGH)	8	25 µg ECpG ODN	14 days of age	10^7^ CFUs of *C*. *jejuni* 15 days of age	37 days of age (22days post-challenge)
5- (LL)	8	Low dose of*C*. *jejuni* lysate	14 days of age	10^7^ CFUs of *C*. *jejuni* 15 days of age	37 days of age (22 days post-challenge)
6- (LH)	8	High dose of *C*. *jejuni* lysate	14 days of age	10^7^ CFUs of *C*. *jejuni* 15 days of age	37 days of age (22 days post-challenge)

UC = unchallenged, untreated control; CC = *C*. *jejuni*-challenged; ECpGL = a low dose (5 µg) of encapsulated CpG ODN; ECpGH = a high dose (25 µg) of ECpG ODN; LL = a low dose (4.3 µg protein) of *C*. *jejuni* lysate (LL); LH = a high dose (21.5 µg protein) of *C*. *jejuni* lysate (LH).

### DNA extraction and 16S rRNA gene sequencing and processing

Microbial genomic DNA extraction was performed using QIAamp DNA Stool mini kit (Qiagen, Toronto, Canada) according to manufacturer’s instructions. DNA concentrations were measured with a Qubit fluorometer (Life Technologies, Eugene, OR), while DNA quality was assessed with a Nanodrop 1000 spectrophotometer (Thermo Scientific, Wilmington, DE). The 16S rRNA gene was PCR-amplified and sequenced on Illumina MiSeq (Illumina, San Diego, CA) using a dual-indexing strategy for multiplexed sequencing developed at the University of Guelph’s Genomics Facility, Advanced Analysis Centre (Guelph, Ontario, Canada) as described previously^42^. Sequences were curated using Mothur v.1.36.1 as described in the MiSeq SOP^43^. Processing of sequences and data analysis were performed as described previously^44^. Briefly, after generation of contigs, and merging of duplicate sequences using the unique seqs command, non-redundant sequences were aligned to trimmed and customized references of the SILVA 102 bacterial database^45^ using the align.seqs command. Then, the unique.seqs command was used to create non-redundant sequences of the aligned reads followed by the removal of chimeric sequences using the chimera.uchime^46^ and remove.seqs commands. Binning of sequences into operational taxonomic units (OTUs) using the nearest neighbor algorithm with 3% dissimilarity was performed with the cluster.split command (taxlevel = 5, cutoff = 0.07), followed by conversion to. shared format using the make.shared command. The sub.sample command in Mothur was then used to ensure the same number of sequences (n = 125355), for each sample. Taxonomy was also assigned to each sequence using the Ribosomal Database Project (RDP) bacterial taxonomy classifier. All OTU-based analyses were performed in Mothur. Alpha diversity was performed in Mothur and analysis of significant difference (P < 0.05) was using Tukey’s post hoc test. Beta-diversity was performed in Mothur, and NMDS was performed with Bray Curtis dissimilarities with analysis of molecular variance (AMOVA) performed to assess the significant difference in the special separation among treatments. Identification and visualization of taxa with differential abundance was performed using the linear discriminant analysis (LDA) effect size (LEfSe) method for high-dimensional class comparisons with a particular focus on metagenomic analyses. Treatment groups were assigned as comparison classes and LEfse identified features that were statistically different between treatments were then compared using the non-parametric factorial Kruskal-Wallis (KW) sum-rank test and Linear Discriminant Analysis (LDA) >2^47^.

## Electronic supplementary material

Supplementary information
