# Supplementary material for: PLGA-encapsulated CpG ODN and Campylobacter jejuni lysate modulate cecal microbiota composition in broiler chickens experimentally challenged with C. jejuni
Source: Sci Rep. 2018 Aug 13;8:12076. doi: 10.1038/s41598-018-30510-w (PMC6089921; doi:10.1038/s41598-018-30510-w)
Supplement: Supplementary file 1 — Supplementary information [file 41598_2018_30510_MOESM1_ESM.pdf]

PLGA-encapsulated CpG ODN and *Campylobacter jejuni* lysate modulate cecal microbiota composition in broiler chickens experimentally challenged with *C. jejuni*

Khaled Taha-abdelaziz[1, 2,*], Alexander Yitbarek[1*], Tamiru Negash Alkie[1,3], Douglas C. Hodgins[1], Leah Read[1], J. Scott Weese[1], Shayan Sharif[1#]

[1]Department of Pathobiology, Ontario Veterinary College, University of Guelph, Guelph, ON, Canada N1G 2W1

[2]Pathology Department, Faculty of Veterinary Medicine, Beni-Suef University, Al Shamlah, 62511, Beni-Suef, Egypt

[3] Present address: Department of Biology, Wilfred Laurier University, Waterloo, ON, N2L 3C5, Canada

[*]These authors have contributed equally.

[#]Corresponding author. Tel.: +1 519 824 4120x54641; fax: +1 519 824 5930.

E-mail address: shayan@uoguelph.ca (S. Sharif).

Figure Legend:

Fig. S1: Box-plots (a) and bar-plots (b) showing the effect of *C. jejuni* challenge compared to unchallenged control (UC) chickens on significant changes in the relative abundance of cecal microbiota at the genus level (Kruskal-Wallis tests, FDR-adjusted $Q < 0.05$).

Fig. S2: Box-plots (a) and bar-plots (b) showing the effect of low (ECpGL) and high dose (ECpGH) compared to PBS-treated, *C. jejuni*-challenged control (CC) chickens on significant change in relative abundance of cecal microbiota at the genus level (Kruskal-Wallis tests, FDR-adjusted $Q < 0.05$).

Fig. S3: Box-plots (a) and bar-plots (b) showing the effect of low (LL) and high dose (LH) of *C. jejuni* lysate-treated compared to PBS-treated, *C. jejuni*-challenged control (CC) chickens on significant changes in relative abundance of cecal microbial communities at the genus level (Kruskal-Wallis tests, FDR-adjusted $Q < 0.05$).

Fig. S1

a

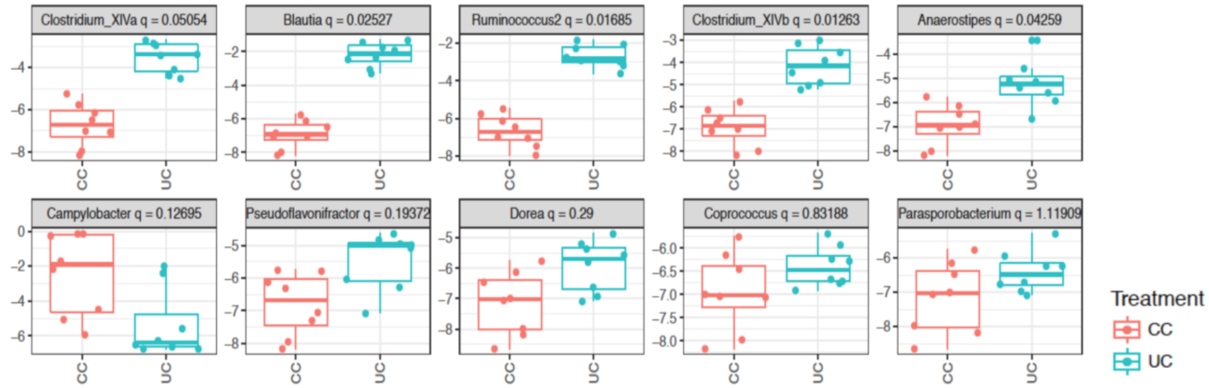

Clostridium_XIVa q = 0.05054
Blautia q = 0.02527
Ruminococcus2 q = 0.01685
Clostridium_XIVb q = 0.01263
Anaerostipes q = 0.04259
Campylobacter q = 0.12695
Pseudoflavonifractor q = 0.19372
Dorea q = 0.29
Coprococcus q = 0.83188
Parasporobacterium q = 1.11909
CC
UC
Treatment
CC
UC

b

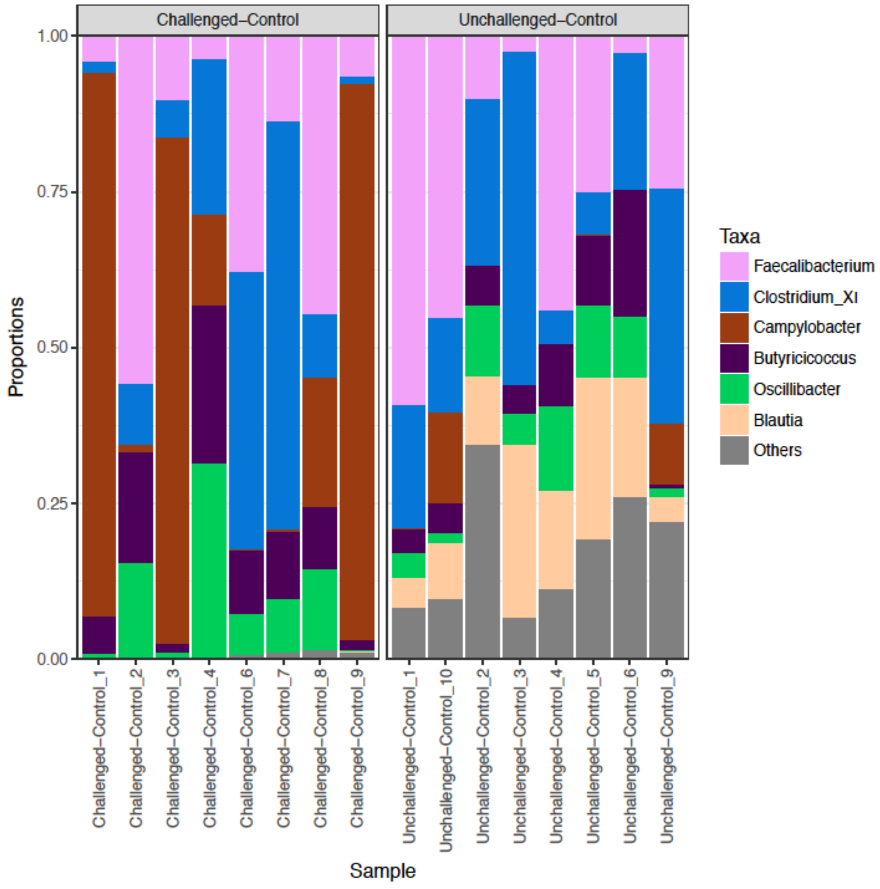

Challenged–Control
Unchallenged–Control
Proportions
1.00
0.75
0.50
0.25
0.00
Taxa
Faecalibacterium
Clostridium_XI
Campylobacter
Butyricicoccus
Oscillibacter
Blautia
Others
Challenged–Control_1
Challenged–Control_2
Challenged–Control_3
Challenged–Control_4
Challenged–Control_6
Challenged–Control_7
Challenged–Control_8
Challenged–Control_9
Unchallenged–Control_1
Unchallenged–Control_10
Unchallenged–Control_2
Unchallenged–Control_3
Unchallenged–Control_4
Unchallenged–Control_5
Unchallenged–Control_6
Unchallenged–Control_9
Sample

Fig. S2

a

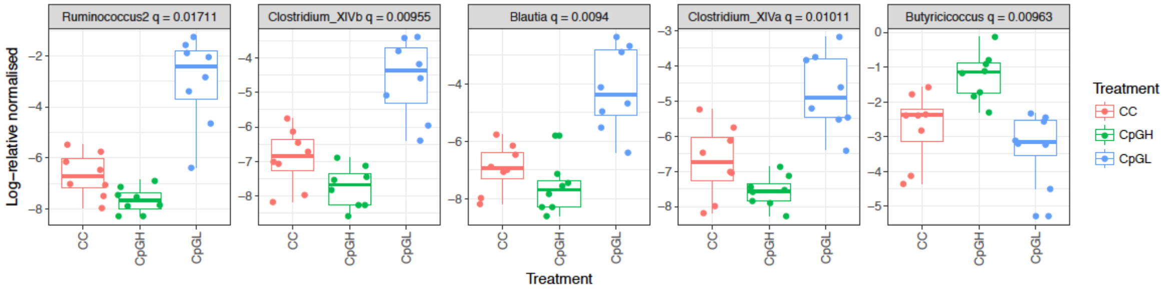
Ruminococcus2 q = 0.01711
Clostridium_XlVb q = 0.00955
Blautia q = 0.0094
Clostridium_XlVa q = 0.01011
Butyricicoccus q = 0.00963
Log−relative normalised
CC
CpGH
CpGL
Treatment
Treatment
CC
CpGH
CpGL

b

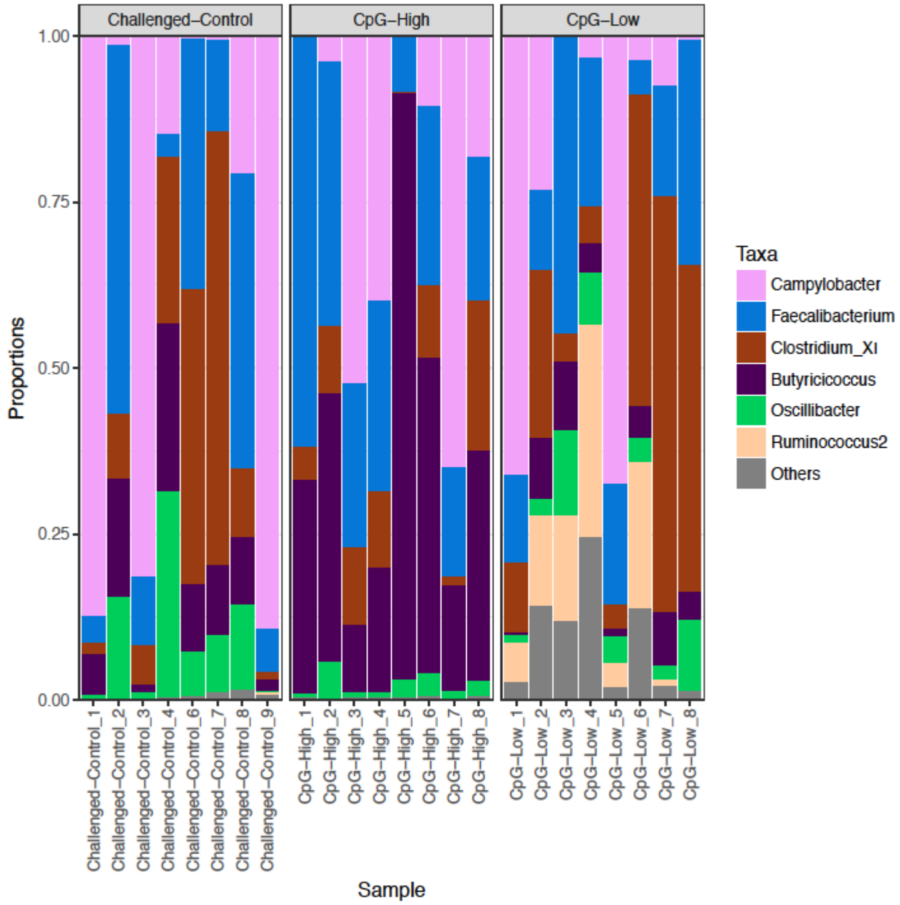
Challenged−Control
CpG−High
CpG−Low
Proportions
1.00
0.75
0.50
0.25
0.00
Sample
Taxa
Campylobacter
Faecalibacterium
Clostridium_XI
Butyricicoccus
Oscillibacter
Ruminococcus2
Others

Fig. S3

a

b

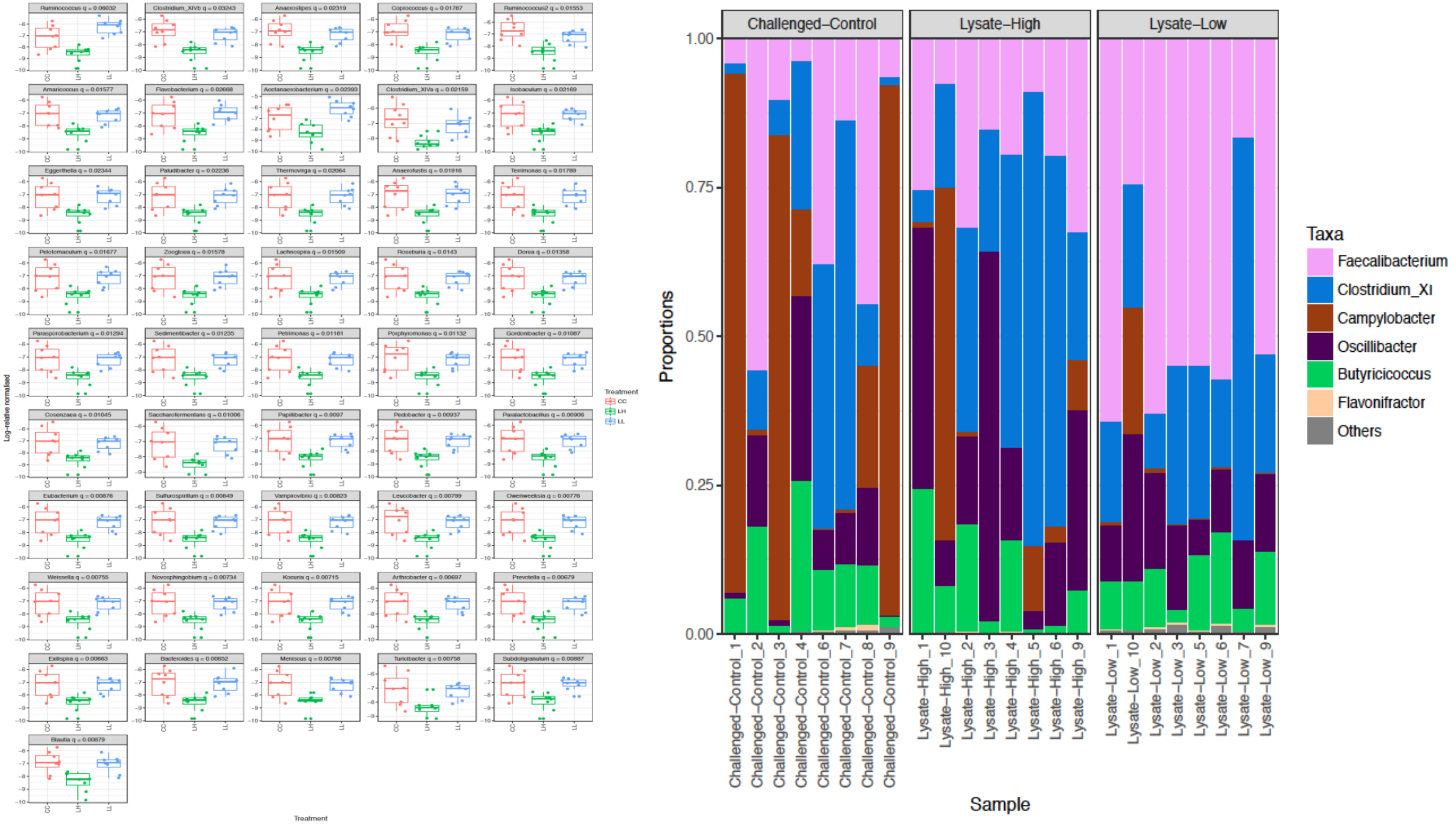
Log-relative normalised
Treatment
CC
LH
LL
Treatment
Challenged–Control
Lysate–High
Lysate–Low
Proportions
1.00
0.75
0.50
0.25
0.00
Sample
Taxa
Faecalibacterium
Clostridium_XI
Campylobacter
Oscillibacter
Butyricicoccus
Flavonifractor
Others
Challenged–Control_1
Challenged–Control_2
Challenged–Control_3
Challenged–Control_4
Challenged–Control_6
Challenged–Control_7
Challenged–Control_8
Challenged–Control_9
Lysate–High_1
Lysate–High_10
Lysate–High_2
Lysate–High_3
Lysate–High_4
Lysate–High_5
Lysate–High_6
Lysate–High_9
Lysate–Low_1
Lysate–Low_10
Lysate–Low_2
Lysate–Low_3
Lysate–Low_5
Lysate–Low_6
Lysate–Low_7
Lysate–Low_9
